# Supplementary material for: Job loss and mental health during the COVID-19 lockdown: Evidence from South Africa
Source: PLoS One. 2021 Mar 30;16(3):e0249352. doi: 10.1371/journal.pone.0249352 (PMC8009396; doi:10.1371/journal.pone.0249352)
Supplement: S1 Appendix — (DOCX) [file pone.0249352.s001.docx]

**S1 Appendix**

Table 6: Proportional odds assumption test

| Regression 1 | | | |
| --- | --- | --- | --- |
|  | Chi2 | df | P> χ^2^ |
| Wolfe Gould | 33.41 | 32 | 0.399 |
| Brant | 33.49 | 32 | 0.395 |
| score | 33.37 | 32 | 0.401 |
| likelihood ratio | 33.65 | 32 | 0.388 |
| Wald | 33.4 | 32 | 0.399 |
| Regression 2 | | | |
|  | Chi2 | df | P> χ^2^ |
| Wolfe Gould | 39.02 | 36 | 0.335 |
| Brant | 39.86 | 36 | 0.303 |
| score | 39.74 | 36 | 0.307 |
| likelihood ratio | 39.63 | 36 | 0.311 |
| Wald | 40.2 | 36 | 0.29 |
| Regression 3 | | | |
|  | Chi2 | df | P> χ^2^ |
| Wolfe Gould | 37.83 | 40 | 0.568 |
| Brant | 39.18 | 40 | 0.507 |
| score | 39.28 | 40 | 0.503 |
| likelihood ratio | 38.59 | 40 | 0.534 |
| Wald | 39.39 | 40 | 0.497 |
| Regression 4 | | | |
|  | Chi2 | df | P> χ^2^ |
| Wolfe Gould | 44.56 | 44 | 0.448 |
| Brant | 48.4 | 44 | 0.3 |
| score | 49.11 | 44 | 0.276 |
| likelihood ratio | 46.74 | 44 | 0.36 |
| Wald | 50.12 | 44 | 0.244 |

Table 7: Marginal Effects, Regressions 1 and 2

|  | Regression (1) | | Regression (2) | |
| --- | --- | --- | --- | --- |
| VARIABLES | Employed  W1 | Employed  W2 | Employed  W1 | Employed  W2 |
|  |  |  |  |  |
| PHQ-2(0) | 0.048** | 0.059** | 0.055** | 0.055** |
|  | (0.023) | (0.024) | (0.025) | (0.026) |
| PHQ-2(1) | -0.003* | -0.003** | -0.003** | -0.003* |
|  | (0.001) | (0.002) | (0.002) | (0.002) |
| PHQ-2(2) | -0.011** | -0.014** | -0.012** | -0.012** |
|  | (0.005) | (0.006) | (0.006) | (0.006) |
| PHQ-2(3) | -0.017** | -0.021** | -0.019** | -0.019** |
|  | (0.008) | (0.008) | (0.009) | (0.009) |
| PHQ-2(4) | -0.008** | -0.010** | -0.009** | -0.009** |
|  | (0.004) | (0.004) | (0.004) | (0.004) |
| PHQ-2(5) | -0.002* | -0.003** | -0.003** | -0.003** |
|  | (0.001) | (0.001) | (0.001) | (0.001) |
| PHQ-2(6) | -0.007** | -0.008** | -0.008** | -0.008** |
|  | (0.003) | (0.003) | (0.004) | (0.004) |
| Observations | 2,213 | 2,213 | 1,941 | 1,941 |

Standard errors in parentheses*** p<0.01, ** p<0.05, * p<0.1

Table 8: Marginal Effects, Regression 3

| Regression (3) | | | | | | |
| --- | --- | --- | --- | --- | --- | --- |
| VARIABLES | Working  W1 | Paid leave  W1 | Furlough  W1 | Working  W2 | Paid leave  W2 | Furlough  W2 |
|  |  |  |  |  |  |  |
| PHQ-2(0) | 0.046* | 0.043 | 0.028 | 0.066*** | 0.091** | -0.016 |
|  | (0.027) | (0.031) | (0.035) | (0.025) | (0.045) | (0.049) |
| PHQ-2(1) | -0.003 | -0.002 | -0.002 | -0.004** | -0.005* | 0.001 |
|  | (0.002) | (0.002) | (0.002) | (0.002) | (0.003) | (0.003) |
| PHQ-2(2) | -0.010* | -0.010 | -0.006 | -0.015** | -0.021** | 0.004 |
|  | (0.006) | (0.007) | (0.008) | (0.006) | (0.010) | (0.011) |
| PHQ-2(3) | -0.016* | -0.015 | -0.010 | -0.023*** | -0.032** | 0.006 |
|  | (0.009) | (0.011) | (0.012) | (0.009) | (0.016) | (0.017) |
| PHQ-2(4) | -0.008* | -0.007 | -0.005 | -0.011** | -0.016** | 0.003 |
|  | (0.005) | (0.005) | (0.006) | (0.004) | (0.008) | (0.009) |
| PHQ-2(5) | -0.002 | -0.002 | -0.001 | -0.003** | -0.004* | 0.001 |
|  | (0.001) | (0.002) | (0.002) | (0.001) | (0.002) | (0.002) |
| PHQ-2(6) | -0.007* | -0.006 | -0.004 | -0.009** | -0.013** | 0.002 |
|  | (0.004) | (0.005) | (0.005) | (0.004) | (0.007) | (0.007) |
| Observations | 2,213 | 2,213 | 2,213 | 2,213 | 2,213 | 2,213 |

Standard errors in parentheses *** p<0.01, ** p<0.05, * p<0.1

Table 9: Marginal Effects, Regression 4

|  | Regression (4) | | | | | |
| --- | --- | --- | --- | --- | --- | --- |
| VARIABLES | Working  W1 | Paid leave  W1 | Furlough  W1 | Working  W2 | Paid leave  W2 | Furlough  W2 |
|  |  |  |  |  |  |  |
| PHQ-2(0) | 0.057** | 0.056 | 0.028 | 0.056** | 0.101** | -0.009 |
|  | (0.029) | (0.034) | (0.037) | (0.027) | (0.049) | (0.055) |
| PHQ-2(1) | -0.004* | -0.003 | -0.002 | -0.003* | -0.006* | 0.001 |
|  | (0.002) | (0.002) | (0.002) | (0.002) | (0.003) | (0.003) |
| PHQ-2(2) | -0.013** | -0.013 | -0.006 | -0.013** | -0.023** | 0.002 |
|  | (0.007) | (0.008) | (0.008) | (0.006) | (0.011) | (0.012) |
| PHQ-2(3) | -0.020** | -0.019 | -0.010 | -0.019** | -0.035** | 0.003 |
|  | (0.010) | (0.012) | (0.013) | (0.010) | (0.017) | (0.019) |
| PHQ-2(4) | -0.010* | -0.010 | -0.005 | -0.010** | -0.017** | 0.002 |
|  | (0.005) | (0.006) | (0.006) | (0.005) | (0.008) | (0.009) |
| PHQ-2(5) | -0.003* | -0.003 | -0.001 | -0.003* | -0.005* | 0.000 |
|  | (0.001) | (0.002) | (0.002) | (0.001) | (0.002) | (0.003) |
| PHQ-2(6) | -0.008* | -0.008 | -0.004 | -0.008** | -0.015** | 0.001 |
|  | (0.004) | (0.005) | (0.005) | (0.004) | (0.007) | (0.008) |
| Observations | 1,941 | 1,941 | 1,941 | 1,941 | 1,941 | 1,941 |

Standard errors in parentheses *** p<0.01, ** p<0.05, * p<0.1

Table 10a: Logit results for binary outcome (PHQ-2) and lag depression score (CESD-10)

|  | (1) | (2) | | | (3) | | (4) | |
| --- | --- | --- | --- | --- | --- | --- | --- | --- |
| VARIABLES | Regression | | Regression | Regression | | Regression | |  |
|  |  | |  |  | |  | |  |
| Binary CES-D 10 score (2017) | -0.041 | | -0.054 | -0.041 | | -0.054 | |  |
|  | (0.128) | | (0.137) | (0.128) | | (0.138) | |  |
| Employed (W1) | -0.315*** | | -0.400*** |  | |  | |  |
|  | (0.120) | | (0.129) |  | |  | |  |
| Employed (W2) | -0.199 | | -0.160 |  | |  | |  |
|  | (0.122) | | (0.132) |  | |  | |  |
| Working (W1) |  | |  | -0.316** | | -0.421*** | |  |
|  |  | |  | (0.138) | | (0.149) | |  |
| Paid leave (W1) |  | |  | -0.314* | | -0.436** | |  |
|  |  | |  | (0.163) | | (0.177) | |  |
| Furlough (W1) |  | |  | -0.183 | | -0.223 | |  |
|  |  | |  | (0.178) | | (0.187) | |  |
| Working (W2) |  | |  | -0.227* | | -0.166 | |  |
|  |  | |  | (0.131) | | (0.140) | |  |
| Paid leave (W2) |  | |  | -0.194 | | -0.185 | |  |
|  |  | |  | (0.230) | | (0.248) | |  |
| Furlough (W2) |  | |  | 0.089 | | 0.085 | |  |
|  |  | |  | (0.244) | | (0.273) | |  |
| Observations | 2,213 | | 1,941 | 2,213 | | 1,941 | |  |

Standard errors in parentheses *** p<0.01, ** p<0.05, * p<0.

Table 10b: Ordered logit results without lag depression score (outcome raw PHQ-2 scores)

|  | (1) | (2) | (3) | (4) |
| --- | --- | --- | --- | --- |
| VARIABLES | Regression | Regression | Regression | Regression |
|  |  |  |  |  |
| Employed (W1) | -0.192** | -0.217** |  |  |
|  | (0.094) | (0.101) |  |  |
| Employed (W2) | -0.238** | -0.220** |  |  |
|  | (0.095) | (0.102) |  |  |
| Working (W1) |  |  | -0.182* | -0.228** |
|  |  |  | (0.107) | (0.116) |
| Paid leave (W1) |  |  | -0.172 | -0.222 |
|  |  |  | (0.126) | (0.136) |
| Furlough (W1) |  |  | -0.112 | -0.113 |
|  |  |  | (0.139) | (0.147) |
| Working (W2) |  |  | -0.265*** | -0.224** |
|  |  |  | (0.101) | (0.109) |
| Paid leave (W2) |  |  | -0.367** | -0.408** |
|  |  |  | (0.182) | (0.196) |
| Furlough (W2) |  |  | 0.064 | 0.035 |
|  |  |  | (0.198) | (0.220) |
| Observations | 2,213 | 1,941 | 2,213 | 1,941 |

Standard errors in parentheses *** p<0.01, ** p<0.05, * p<0.1

Table 10c: Regression (OLS) results for normalized outcome (raw PHQ-2 scores) and normalized lag depression score (CES-D10)

|  | (1) | (2) | (3) | (4) |
| --- | --- | --- | --- | --- |
| VARIABLES | Regression | Regression | Regression | Regression |
|  |  |  |  |  |
| Normalized CES-D 10 score (2017) | 0.022 | 0.019 | 0.022 | 0.019 |
|  | (0.022) | (0.024) | (0.022) | (0.024) |
| Employed (W1) | -0.119** | -0.141** |  |  |
|  | (0.051) | (0.055) |  |  |
| Employed (W2) | -0.124** | -0.118** |  |  |
|  | (0.052) | (0.055) |  |  |
| Working (W1) |  |  | -0.112* | -0.143** |
|  |  |  | (0.058) | (0.063) |
| Paid leave (W1) |  |  | -0.107 | -0.148** |
|  |  |  | (0.068) | (0.073) |
| Furlough (W1) |  |  | -0.063 | -0.067 |
|  |  |  | (0.075) | (0.079) |
| Working (W2) |  |  | -0.145*** | -0.129** |
|  |  |  | (0.055) | (0.059) |
| Paid leave (W2) |  |  | -0.159* | -0.182* |
|  |  |  | (0.095) | (0.102) |
| Furlough (W2) |  |  | 0.092 | 0.092 |
|  |  |  | (0.107) | (0.118) |
| Observations | 2,213 | 1,941 | 2,213 | 1,941 |

Standard errors in parentheses *** p<0.01, ** p<0.05, * p<0.1

Note: Regressions in 10 a, b and c include the full set of covariates.
