# Supplementary material for: Job loss and mental health during the COVID-19 lockdown: Evidence from South Africa
Source: PLoS One. 2021 Mar 30;16(3):e0249352. doi: 10.1371/journal.pone.0249352 (PMC8009396; doi:10.1371/journal.pone.0249352)
Supplement: Attachment — Submitted filename: response to reviewers.docx [file pone.0249352.s002.docx]

**Job loss and depression: Responses to reviewers**

We thank the reviewers for their comments and suggestions, many of which we have implemented, and which have improved the quality of the paper. Where we have not made changes, we have explained our reasons. Detailed responses are below.

**Journal requirements**

**1. Formatting and file labelling:**

**2. Please include a copy of Tables 4 and 5 which you refer to in your text on page 13.**

- These tables are included at the end of the “Results” section, on pages 15 and 16.

**Reviewer 1**

**Major comments**

**(1)** **Contribution and related literature**: **Recent published work has tried to understand how changes in mental wellbeing between pre-COVID and COVID periods can be related to being employed or being furloughed** (Banks and Xu, 2020): <https://onlinelibrary.wiley.com/doi/full/10.1111/1475-5890.12239>. **The current paper must frame its methodology, limitations and findings in the context of what has already been done and is already known. How do the methodology and main findings compare with recent COVID studies on employment and mental health? For instance, how do the PHQ-2 and the GHQ-12 measures (the GHQ-12 is used in Banks and Xu, 2020) compare when measuring mental health?**

Thank you for this reference, which we now cite in the study. However, in contrast to Banks and Xu (2020), our study does not investigate how mental health changed from the pre-COVID to the COVID periods. Rather, we use the unprecedented job loss that followed the lockdown to explore the implications of job loss for the mental health of a sample of adults who were employed before lockdown. i.e. We use the response to COVID (the lockdown) as a natural experiment, which allows us to overcome the typical problems that arise when exploring the relationship between job loss and mental health viz. that job loss may be endogenous to mental health. This is also the first study that focuses on the implications of job loss for mental health in South Africa, a country with very high rates of unemployment, and where the mental health implications of unemployment are not adequately recognized in public policy.

In the conclusion, we now recognize that the PHQ-2 is an attractive measure of vulnerability to depression when there are stringent constraints on the length of the instrument, as has been the case for data collection during COVID. But we recognize also that it offers only an initial measure of depressive symptoms, and that in contrast to other measures such as the PHQ-9 and the CES-D 10, it will be less sensitive to variation or severity in mental health.

**(2) Selective attrition: Does mental health at baseline predict participation in W1 and W2?**

A simple test of attrition using a probit model (Fitzgerald et al. 1998) shows that the probability of not being sampled in NIDS-CRAM Wave 1 is not related to depression scores at baseline (in 2017) (the estimated coefficient = -0.0006 with s.e. = 0.002). The probability of attrition from Wave 1 of NIDS-CRAM to Wave 2 is also not significantly associated with mental health at baseline (coefficient = -0.003, se = 0.005) i.e. There is no evidence of attrition into NIDS-CRAM, or through NIDS-CRAM, based on mental health. We have now recognized this in the section, “Data and methods”.

**(3) Comparability of measures of mental health: What do we know about the relationship between the PHQ-2 and the CES-D 10 from previous studies? What is the fraction of individuals with PHQ>=3 and CES D -10 >=10 in previous studies?**

The study explores the association between job loss and depression scores, as measured by PHQ-2. We could not see why the relationship between the PHQ-2 and the CES-D 10 scores would be relevant for the study. In our estimations, we do not directly compare the CES-D 10 scores from 2017 with the PHQ-2 scores from 2020, to draw inferences about how depression scores have changed over time i.e*.* We are not measuring a change in depression scores from pre-COVID times to COVID times. We also use the scores as a continuous index, rather than imposing thresholds to identify depression. We only include CES-D 10 scores from 2017 as a covariate in the estimations (and therefore estimate lagged models) to provide some control for an unobservable “propensity” to report or experience depressive symptoms. We have now deleted the following sentence from the discussion section, in case this was misleading:

“To the extent that depression scores derived from different instruments can be compared, descriptive analysis identified a higher incidence of depression among the study’s sample in 2020 (24%), compared to 2017 (17%).”

**(4) Sample: The study focuses on all adults who were employed in the month before the COVID-lockdown started. Given that the focus is on employment status, it would be more appropriate to focus on a more restricted age group: 18-64. Currently, the maximum age (Table 1) is 89!**

The sample includes only those ‘elderly’ who had been employed before lockdown. We chose to analyse the full sample of adults with prior employment for two reasons. At a conceptual level, we did not want to assume that losing employment would not affect the mental health of those who are beyond age 60 (when people are age-eligible for a state pension) or beyond age 64. Many adults who are outside of the working-age range still need to work in South Africa given insufficient savings and retirement benefits. At a practical level, given that the sample was relatively small, we wanted to maximize the sample size for the analysis.

**5) Sampling weights: The authors do not mention the use of sampling weights, but they should explain why they do not use the available NIDS (-CRAM) sampling weights. If there is no reason that justifies their choice, I am afraid that sampling weights must be used.**

We have given considerable thought to whether the analysis should use the weights. Our concern is that the weights are benchmarked to a sample in 2017, which, as a fifth wave of a panel, was itself not nationally representative. Our sample is also restricted to those who were employed before lockdown, and the weights are not stratified by employment status. We therefore think that a model-based approach is more suited in the context of this study (Winship and Radbill 1994). We have added this explanation in the text and are careful throughout the study to refer only to sample estimates.

(**6) The discussion section can be broken down into two sections: “discussion” and a “conclusion”. Pros and cons of the study must be clearly acknowledged. Cons of the study include the fact that not only mental health is measured on a different scale before the pandemic, but a different collection method is used. This should probably be discussed, if not accounted for.**

Thank you for these comments. We have now separated the discussion section as suggested, and we have acknowledged some of the pros and cons of the study in the conclusion. But note that the study does not compare depression scores before the pandemic and during the pandemic, precisely because of the limitations which are highlighted in this comment (that mental health is measured on a different scale and was collected in a different way).

**(7) The finding on race/ethnicity (African vs. non-African) is intriguing. Recently, Proto and Quintana-Domeque (2020) show that in the UK there are differences by gender and ethnicity in the deterioration in mental health between pre-COVID and COVID periods. Given the previous research by Proto and Quintana-Domeque (2020) and the findings in this paper, the authors should include the interaction between gender and race. In addition, it is standard practice to define gender as female (and the reference category as male), so that the new regressions should include the following three dummies: Female, African, and Female*African**.

Thank you for this suggestion. We have now changed the reference category to male. We re-estimated our models with the interactive dummy (female*African) included, but the results are not significant in any of the estimations and so we have not included the interaction term i.e. There is no evidence that vulnerability to depression during COVID differs by gender and ethnicity. We have recognized this at the end of the discussion section, adding Proto and Quintant-Domeque (2020) to the references.

**Minor comments**

**a. The title of the manuscript seems a bit misleading: the authors acknowledge that they cannot control for individual fixed effects since they do not observe the dependent variable before the pandemic. This limitation seems important when presenting their evidence as longitudinal**.

- The outcome variable is cross-sectional, but we do use longitudinal data in the estimations, by distinguishing employment status in each of the two waves. We also incorporate longitudinal data from 2017 by controlling for depression scores in 2017. The lagged models require panel data. Nonetheless, we have now removed “longitudinal micro-data” from the title.

**b. The numbering of tables is not correct. The text refers to Table 3 (p.11), but indeed, there is Table 2 and then “Table 1”, “Table 2” and Table 3” reporting the marginal effects for regressions 1-2, 3 and 4, respectively. Similarly, the text refers to Table 5 (p.13), but there is no Table 5**.

- Our apologies, the tables in the appendix were incorrectly numbered, which we have now corrected. The tables within the body of the paper are correctly numbered and complete.

**c. Typos: Table 1 reports a mean of 10.184 for the binary variable “Coronavirus can be avoided”. This should be fixed.**

- Thank you for spotting this. We have now corrected the mean value.

**d. Pages 9-10: Equations are not numbered. Moreover, the variables y*, x and Y should be indexed with i, and the cut-off mu should not be indexed with i, but with a different letter.**

- We have made these changes.

**e. Page 10: The authors write “An insignificant outcome indicates that the assumption has been met.” A more precise statement follows: “An insignificant outcome indicates that** **there is not enough strong evidence against the parallel regression assumption.”**

- Thank-you, we have made this correction.

**f. Some of the findings are statistically significant at the 10% level (e.g. Social grants): “The claim that the regression analysis pointed to the importance of social grants (or cash transfers) in providing protection against the incidence of depressive symptoms” seems too strong. The causal language should be tuned down and the authors should not emphasize statistically significant findings at the 10% level.**

- We reported on a lower significance threshold in this paper than usual (i.e. the 10% level), given small sample sizes when we disaggregate into sub-groups. But we have moderated the discussion, recognising that associations at the 10% level are only weakly significant.

**References

• Banks, J. and Xu, X. (2020) “The Mental Health Effects of the First Two Months of Lockdown during the COVID-19 Pandemic in the UK,” Fiscal Studies:** <https://onlinelibrary.wiley.com/doi/full/10.1111/1475-5890.12239>

**• Proto, E. and Quintana-Domeque, C. (forthcoming) “COVID-19 and mental health deterioration by ethnicity and gender in the UK”, PLOS ONE. Previous version:** <https://www.iza.org/publications/dp/13503/covid-19-and-mental-health-deterioration-among-bame-groups-in-the-uk>

- These are now included in the paper.

Reviewer 2

**Reviewer #2: 1. Is the manuscript technically sound, and do the data support the conclusions?
The authors have gone to painstaking lengths to eradicate nearly all concerns about the causal relationship between job loss, furlough, and mental health. Starting with the basics of making a case for an exogenous relationship between job loss and mental health, to noting the sampling to minimize bias found in typically online-only sampling (for rapid sampling, the authors have done an impressive job), to drawing upon a well-established nationally representative survey, to the missingness of data and being transparent that the data appear missing at random based on observed covariates (a scientifically transparent and important distinction from truly missing at random or missing completely at random), to disclosing the inability to account for individual fixed effects pertaining to influences on mental health. I expect that the scholarly community reading this paper may take issue with the exogeneity argument, particularly as those at the margins of the workforce in South Africa—in very tenuous employment, of which many Black South Africans are in—who could have been on the verge of losing their jobs anyway. Nonetheless, this is about as good as one could get for research design and assessing cause and effect. The conclusions drawn are very appropriately rooted in the methods and data.**


**2. Has the statistical analysis been performed appropriately and rigorously?
In short, yes.**

**The CES-D 10 is appropriate and externally (and within South Africa among different racial/ethnic groups) validated for measuring depression. The regression models are soundly developed and the authors clearly have not taken any shortcuts—little things like adjusting for age by also including the quadratic are very understated but key to making this research technically sound). Further, noting the parallel regression assumption and how they adjusted for that in their programming is generally considered “best practices” and something that most scholars take for granted/do not discuss when presenting ordered logistic regression models.

I am surprised that the results are not stratified by race/ethnicity as this moderates nearly everything in South Africa. Another suggestion would be to specify your variable coding a bit more. The average reader may be confused by the African versus non-African distinction and what it means in South Africa—I too am a bit confused mainly because I would like to know which category Coloured South Africans fall into (I also recognize that Indian and White South Africans account for only a small share of the overall population). The tables seem out of order or out of place in the manuscript too. The results are all there but the table numbering is off.**

- Thank you, we have corrected the tabling numbering.

- Non-African includes Coloureds, Indians and Whites. We have now specified this in the text. We do not distinguish among these groups in the estimations as the coefficients are all individually insignificant (reflecting, at least in part, low sample numbers).

.
