# Supplementary material for: Job loss and mental health during the COVID-19 lockdown: Evidence from South Africa
Source: PLoS One. 2021 Mar 30;16(3):e0249352. doi: 10.1371/journal.pone.0249352 (PMC8009396; doi:10.1371/journal.pone.0249352)
Supplement: Attachment — Submitted filename: Response to reviewer_second set.docx [file pone.0249352.s003.docx]

Reviewer #1: Report on PONE-D-20-38028R1: "Job loss and mental health during the COVID-19 lockdown: Evidence from South Africa"

Thank you for addressing my previous points.

I think the paper is almost ready for publication. My requested minor revision consists in the following two extensions:

(1) Additional Table 2 for binary dependent variable: Depression (0-1)

The model to be run is:
D(t) = a + bX + cD(t-1) + error term,
where D(t) and D(t-1) are the indicators of depression at t and t-1:
D(t) = 1 if PHQ2 >=3, D(t) = 0 if PHQ2 <3
based on [Kroenke et al. 2003] as discussed on p. 11,
and D(t-1) = 1 if CES-D 10 >=10, D(t-1) = 0 if CES-D 10 < 10

based on [33] as discussed on p. 11.

(2) Additional Table 2 for change in binary dependent variable: Change in Depression

The model to be run is:

D(t) - D(t-1) = a + bX + error term

Required assumption: "Depression scores derived from different instruments (PHQ-2 and CES-D 10) can be compared."

Response:

As the reviewer suggested, we have estimated the model with a binary dependent variable for depression at time t, and with a binary covariate of depression in time t-1. Our results remain robust, although some coefficients (e.g. for employment status in period 2) lose significance in some of the regressions.

However, as we explain in the data and methods section of the paper (p.8), the appropriate cut-off for depression in South Africa has been found to vary across the different language groups (as a proxy also for ethnicity) in the country (Baron et al. 2017). Our preferred method therefore is to follow the approach adopted in other studies which have analysed South African micro-data on depression and to use the scales to capture a continuum of depression. (See for example, studies by Ardington and Case 2010; Tomita and Burns 2013; Meffert et al. 2015; Burger et al. 2017.)

We were also unsure about the motivations for the suggestion to model a change in the likelihood of depression (using the different instruments) as a function of conditions only in time t. Our intention, in including a “baseline” regression score as a covariate, was only to offer some control for a “predisposition” to experience depressive symptoms or to an idiosyncratic over-/under-reporting of depressive symptoms. We therefore attach no weight to a comparison of the different depression scales from 2017 and 2020. We have now been explicit in the methods section (page 7) that robust conclusion about depressive symptoms pre-COVID in 2017 and during COVID in 2020 cannot be drawn.

If the concern is with the inclusion of the CES-D 10 scale as a covariate in the estimations, then we have estimated two further robustness checks:

- First, we estimated the pooled model without the CES-D 10 score as a covariate.

- Second, we normalised the PHQ-2 scores and the CES-D 10 scores and re-estimated the pooled model.

Our results in these models are all consistent with the results reported in the paper although some of the coefficients in the binary model lose significance. We have included these robustness checks and the binary model in the Appendix (see Tables 10a - c), providing reference to these checks in the results section (pp.13 and 14)

Finally, proofreading is required. One example:

-Kroenke et al (2003) [https://pubmed.ncbi.nlm.nih.gov/14583691/] is missing from the reference list.
-NB. Kroenke et al (2003) on p.11 should be displayed as a [number] not as a name.

Response:

Thanks for pointing this out – we have converted this and checked other references, and we have proofread the paper more generally.
